# Delirium in ICU Patients after Cardiac Arrest: A Scoping Review

**DOI:** 10.3390/jpm12071047

**Published:** 2022-06-27

**Authors:** Wioletta Mędrzycka-Dąbrowska, Sandra Lange, Dorota Religa, Sebastian Dąbrowski, Adriano Friganović, Ber Oomen, Sabina Krupa

**Affiliations:** 1 Department of Anaesthesiology Nursing & Intensive Care, Faculty of Health Sciences, Medical University of Gdansk, Dębinki 7, 80-211 Gdańsk, Poland; 2 Department of Internal and Pediatric Nursing, Medical University of Gdańsk, Dębinki 7, 80-211 Gdańsk, Poland; langa94@gumed.edu.pl; 3 Department of Neurobiology, Care Sciences and Society (NVS), Karolinska Institutet, 17177 Stockholm, Sweden; dorota.religa@ki.se; 4 Departament of Medical Rescue, Faculty of Health Sciences, Medical University of Gdańsk, Dębinki 7, 80-211 Gdańsk, Poland; sebastian.dabrowski@gumed.edu.pl; 5 Department of Anesthesiology and Intensive Medicine, University Hospital Centre Zagreb, 10000 Zagreb, Croatia; adriano@hdmsarist.hr; 6 Department of Nursing, University of Applied Health Sciences, Mlinarska Cesta 38, 10000 Zagreb, Croatia; 7 European Specialist Nurses Organisation (ESNO), 6821HR Arnhem, The Netherlands; secretariat@esno.org; 8 Institute of Health Sciences, College of Medical Sciences of the University of Rzeszow, St. Warzywna 1A, 35-310 Rzeszow, Poland; sabinakrupa@o2.pl

**Keywords:** delirium, cardiac arrest, resuscitation, ICU, a scoping review

## Abstract

Introduction: The incidence of delirium in the intensive care unit is high, although it may differ according to the specific characteristics of the unit. Despite the rapid development of research on delirium in recent years, the pathophysiological mechanisms leading to the clinical presentation of delirium are still subject to hypotheses. The aim of this review was to describe the incidence of delirium in cardiac arrest survivors and the clinical impact of delirium on patient outcomes. Methods: A scoping review was conducted in the second quarter of 2022. The number of articles retrieved during each search test was limited to studies conducted between 2010 and 2020. Strict inclusion and exclusion criteria were applied. The last search was conducted in May 2022. Results: A total of 537 records was initially obtained from the databases. After discarding duplicates, selecting titles and abstracts, and then analyzing full-text articles, 7 studies met the inclusion criteria. The incidence of delirium in the cardiac arrest survivor population ranged from 8% to as high as 100%. The length of stay in ICU and hospital was significantly longer in patients with delirium than those without. Ninety-eight percent of patients had cognitive or perceptual impairment and psychomotor impairment. Of the seven studies included in the analysis, the RASS, CAM, and NuDesc scales were used to diagnose delirium. Potential risk factors that may influence the duration of delirium include age and time since resuscitation; propofol use shortened the duration of delirium. Conclusion: the incidence of delirium in ICU patients who survived CA is high. Cardiac arrest is an additional predisposing factor for delirium. In cardiac arrest survivors, the occurrence of delirium prolongs the duration of ICU and hospital stay and adversely affects functional outcomes. The most common type of delirium among this population was hypoactive delirium. A large percentage of patients manifested symptoms such as cognitive or perception impairment, psychomotor impairment, and impaired concentration and attention.

## 1. Introduction

Over the past few years, medical advances, including targeted temperature control, have increased the rate of successful resuscitation and survival after cardiac arrest. Cardiac arrest, initial resuscitation efforts, and post-resuscitation management affect the possibility of recovery and the risk of delirium [1]. Data suggest that delirium in cardiac arrest survivors is an independent risk factor for morbidity and mortality [1,2]. The diagnosis of delirium in patients with cardiac arrest can be difficult due to the differentiation between encephalopathy, primary neurological damage, and delirium [1]. According to a meta-analysis by Salluh et al., delirium may affect up to 80% of patients admitted to the intensive care unit (ICU) and according to Polloc et al. the proportion is up to 100% in patients after cardiac arrest treated with mild therapeutic hypothermia [3,4]. Identification of modifiable risk factors, early recognition of symptoms, and initiation of coordinated treatment strategies can help to predict adverse outcomes [1]. The onset of delirium is often multifactorial. Post-cardiac arrest brain injury (PCABI) is caused by initial ischemia and subsequent reperfusion of the brain following resuscitation [5,6]. Hepatic and renal function may be impaired in more than 50% of patients after cardiac arrest, so pharmacokinetic changes in administered drugs can be significant [7]. In critically ill patients, delirium is associated with higher mortality, prolonged hospitalization, and an increased risk of cognitive impairment; i.e., disorientation, deficits in attention, memory, thinking, anxiety, psychomotor agitation, hallucinations, disturbances of the sleep-wake cycle and affective symptoms, and disorganization [1,5]. Patients after cardiac arrest are classically excluded from studies of delirium. This results in a lack of information about the phenotype, risk factors, and optimal treatment of delirium after cardiac arrest [7]. Despite the rapid increase in the number of studies on delirium in recent years, the pathophysiological mechanisms leading to the clinical presentation of delirium are still hypothesized [7,8,9,10,11]. It is noteworthy that different ICU care processes affect delirium and have an impact on patients after cardiac arrest [4]. The purpose of this scoping review was to collate the information that has been published on delirium that has occurred in patients after cardiac arrest. A scoping review is used to determine the scope or extent of the literature on a new topic and aims to identify available evidence to inform the review and guide further research [12]. Given the limited number of studies published on this topic, the scoping review methodology was most appropriate for the purposes of this review. This review can inform future research to answer more precise questions and resolve identified research questions.

### Aim

The aim of the review was to describe the incidence of delirium in cardiac arrest survivors and the clinical impact of delirium on patient outcomes.

## 2. Methods

### 2.1. Study Design

The scoping review was conducted in the second quarter of 2022. Scoping reviews are a relatively new approach to synthesizing evidence, and there is currently little guidance on deciding between a systematic review and a scoping approach during the synthesis of evidence, especially when the literature has not yet been comprehensively reviewed or shows a large, complex, or heterogeneous nature that cannot be subject to a more thorough systematic review [12].

### 2.2. Review Questions

To identify important aspects related to delirium in patients after cardiac arrest, we developed research questions that clearly define the population, concept, and context (PCC) of the scoping review.

1. What is the incidence of delirium in patients after cardiac arrest?
2. Is cardiac arrest associated with higher risk of delirium and what is the impact on delirium duration?
3. What impact does delirium after cardiac arrest have on patient outcomes?
4. What a type of delirium occurs in patients after cardiac arrest?

### 2.3. Search Strategy

Two authors systematically searched the following databases: PubMed, EBSCO, Web of Science, and Cochrane Library. The following keywords were used: “ICU”, “intensive care”, “delirium”, “cardiac arrest”, “resuscitation”, “delirium after cardiac arrest”, “delirium after CPR”. Keywords were entered along with their combinations using AND or OR. All publications were analyzed by title and abstract to exclude irrelevant entries. Secondly, a manual internet search with Google Scholar was performed. Any discrepancy was resolved through discussion with the four researchers, and at the end of the selection process, full agreement was reached on the articles to be included. Data including author (first), target, participants, interventions, results, and findings were extracted from all eligible studies. The number of articles found during each search test was limited to surveys conducted between 2010 and 2020. The initial search was conducted from early February to May 2022 and the final search was carried out on 20 May 2022. To identify relevant studies, we used the population−concept−context (PCC) framework recommended by the Joanna Briggs Institute (JBI) [13]. Strict inclusion and exclusion criteria were applied (Table 1). Reviews are considered eligible if all the following criteria are met.

### 2.4. Study Selection

Following the PCC framework, our scoping review included research reporting data on adult ICU patients (>18 years) (P) diagnosed with delirium (C) that occurred after cardiac arrest (C). We excluded studies whose participants were children (<18 years), non-ICU patients with undiagnosed delirium and where delirium did not occur after cardiac arrest. We also excluded publications in a language other than English and articles for which the full version could not be accessed.

### 2.5. Data Extraction

Data extraction, which is referred to in the scoping review as ‘data charting’ [13,14] was undertaken by two reviewers independently. Information extracted from included studies included first author’s name, year of publication, study design, participants, delirium assessment tool, number of delirium incidents after CA, and findings. The authors performed the extraction using Microsoft Excel.

### 2.6. Assessment of Study Quality of the Included Studies

The Joanna Briggs Institute (JBI) critical appraisal checklist was used to assess the methodological quality of the study and study possibility of bias in its design, conduct, and analysis [15]. The assessment process are presented in Table 2.

Q1.Were the two groups similar and recruited from the same population? Q2. Were the exposures measured similarly to assign people to both exposed and unexposed groups? Q3. Was the exposure measured in a valid and reliable way? Q4. Were confounding factors identified? Q5. Were strategies to deal with confounding factors stated? Q6. Were the groups/participants free of the outcome at the start of the study (or at the moment of exposure)? Q7. Were the outcomes measured in a valid and reliable way? Q8. Was the follow up time reported and sufficient to be long enough for outcomes to occur? Q9. Was follow up complete, and if not, were the reasons to loss to follow up described and explored? Q10. Were strategies to address incomplete follow up utilized? Q11. Was appropriate statistical analysis used?; 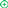-Yes; 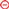-No; n/a-not applicable.

## 3. Results

**Figure 1:**
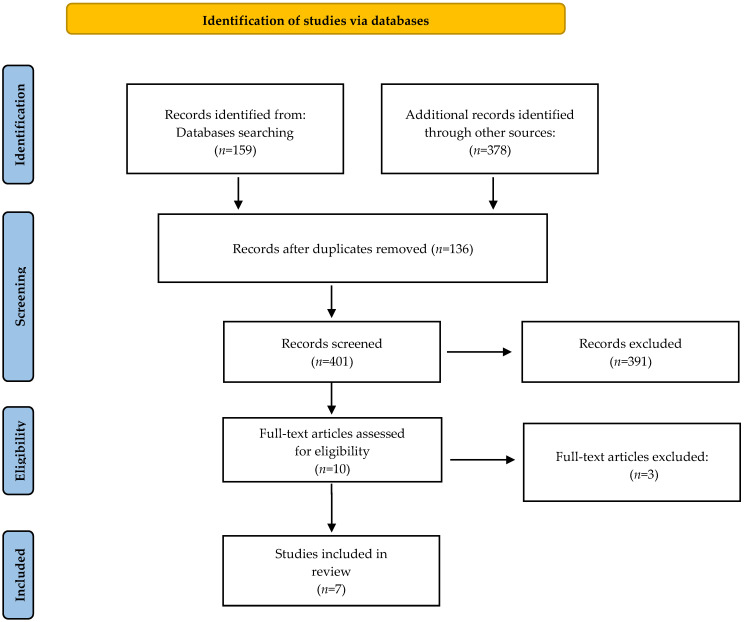
PRISMA flow diagram [15].

A total of 537 records was initially obtained from the databases: PubMed—78, EBSCO—2, Web of Science—53, Cochrane Library—26, and Google Scholar—378. After discarding duplicates and selecting titles and abstracts, 527 were excluded, leaving 10 articles of full text that were analyzed. Of these, 3 were excluded for failing to meet the inclusion criteria. Seven reviews met the inclusion criteria [2,4,16,17,18,19,20]. The results are presented in Figure 1. Table 3 presents a summary of the main results of the scoping review.

### 3.1. Incidence of Delirium in Patients after CA

Of the seven studies included in the analysis, three used the RASS and CAM scales to diagnose delirium, two were assessed by a psychiatrist according to DSM scale criteria, and in one delirium was diagnosed using the RASS scale and NuDesc. Most participants were hospitalized in the cardiac intensive care unit (CICU). The incidence of delirium in the cardiac arrest survivor population ranged from 8% in the Falsini et al. study [18] to up to 100% in the Pollock et al. study [4]. In the Pauley et al. study, delirium after cardiac arrest occurred in 21/120 (18%) [19], followed by Jäckel et al., 15/68 (22%) [16], Rezar et al., 24/106 (23%) [16], Uguz et al., 3/12 (25%) [20], and Keijze et al., 47/141 (33%) [2].

### 3.2. Cardiac Arrest as a Predictor of Delirium

In a subgroup of patients from the study by Jäckel et al. staying in the ICU > 24 h, multivariable logistic regression analysis showed that cardiac arrest was one of the predictors of delirium in the study group [17]. In a study by Pauley et al., 31 people who were admitted to the intensive care unit were post-cardiac arrest [19]. Of these, 21 (18%) were diagnosed with delirium [6]. In the group of patients in the Uguzet et al. study, 12 patients were diagnosed with delirium. Of these, three survived cardiac arrest during myocardial infarction (25%). Logistic regression analysis by Uguzet al. showed that survival of cardiac arrest during myocardial infarction was an independent predictor of the development of delirium [20].

### 3.3. Impact of Delirium in Patients after CA on Outcomes

Only one study analyzed the effect of delirium on neurological outcomes, length of ICU and hospital stay in patients after CA. In the study by Keijze et al., neurological recovery was measured 6 months after cardiac arrest using the Cerebral Performance Category (CPC) scale during a telephone interview. Results showed that the length of stay in the ICU and hospital was significantly longer in patients with delirium. The median (IQR) for length of stay in the ICU was 6(9) days for patients with delirium and 3(4) days for patients without delirium (*p* < 0.01). The median total length of hospital stay was 24(21) days for delirium patients and 15(15) days for non-delirium patients (*p* < 0.01). Furthermore, the analysis showed that patients who developed delirium were more likely to be discharged to chronic nursing homes (15% vs. 4%; *p* = 0.03) or a rehabilitation center (19% vs. 3%, *p* < 0.01). The chance of poor outcome was higher in patients with delirium than without, although the difference was not statistically significant [2].

Pollock et al. investigated potential risk factors that may influence the duration of delirium. Among the factors before resuscitation, multivariable proportional odds logistic regression showed that age (OR 1.72, 95% CI 1.01–2.95, *p* = 0.05) and time from the start of resuscitation to ROSC (OR 1.52, 95% CI 1.11–2.07, *p* = 0.01) were associated with an increased number of days of delirium. In contrast, the use of propofol during therapeutic hypothermia (TH) sedation reduced the duration of delirium (OR 0.02, 95% CI 0.00–0.48, *p* = 0.02) [4].

Rezar et al. analyzed gender differences in clinical management and outcomes after cardiac arrest. Their analysis showed no statistically significant difference in the incidence of delirium between men and women (26.3% vs. 13.3%, *p* = 0.200) [16].

### 3.4. Subtype of Delirium

The type of delirium that dominated in patients after CA could only be determined in two studies. Among patients in the Pollock et al. study, most delirium was hypoactive. Hyperactive delirium occurred over a minimum of one or more days in 21% of patients. Sixty-four percent of patients had at least one day of mixed delirium [4]. In the study by Keijze et al., 98% of patients had cognitive or perceptual impairment and psychomotor dysfunction. Seventy-nine percent of patients had impaired concentration and attention. More than half experienced: extreme restlessness (57%), disinhibition (55%), emotional disturbances (55%), and language disorders (51%). Patients also had sleep disorders. Forty-seven percent experienced hallucinations and 43% had sleep−wake cycle disturbances. Other symptoms included: wandering (43%), shouting (30%), aggression (23%), paranoia (19%), head shaking (19%), incontinence (13%), excessive drinking (11%) [2].

## 4. Discussion

The frequency of delirium in the intensive care unit is high, although it may differ depending on the specifics of the unit [4]. It is estimated that the disorder may develop in up to 80% of intensive care patients [21]. Survivors of cardiac arrest are a specific group of patients who are often excluded from studies [2]. Given that there has been an increase in survival rates after cardiac arrest in recent years, this population may represent an increasing proportion of ICU patients [22,23]. Cardiac arrest itself is associated with neurological impairment and an overall poorer prognosis [24,25]. Therefore, cardiac arrest may also be a potential risk factor for delirium and affect patient outcomes [2,17,18,19]. In the study by Pollock et al., each patient (100%) who survived sudden cardiac arrest underwent TH developed delirium that lasted at least one day during their ICU stay [4]. Although this high percentage did not occur in other studies, the incidence of delirium was also relatively high. In a study by Keijze et al., one-third of patients (33%) who recovered from CA had symptoms of delirium [2]. In a retrospective study by Aicher et al. whose results were published in abstract form, delirium occurred in 79/93 patients (84.9%), which confirms the high incidence of delirium in this group of patients [26]. In critically ill patients, the occurrence of delirium is associated with prolonged hospitalization, higher mortality, and a higher incidence of cognitive impairment at discharge [17,18,19,27,28]. Similarly, survivors of cardiac arrest who developed delirium had a higher median length of hospitalization in the ICU and hospital. These patients were also more likely to be discharged to nursing homes or rehabilitation centers than patients who did not experience delirium [2].

The major risk factors for delirium in critically ill patients include old age and pre-existing cognitive impairment. People admitted to the ICU with a diagnosis of myocardial infarction after cardiac arrest, acute respiratory failure, and acute valvular disease were more likely to be positive for delirium on the CAM-ICU scale [19]. This result is consistent with the observations of Uguz et al. and Jäckel et al. [17,20]. Logistic regression analysis by Uguz et al., showed that in patients after acute myocardial infarction, survival of cardiac arrest during myocardial infarction was an independent predictor of the development of delirium [20]. In addition, advanced age, degrees of freedom, and higher potassium levels at admission were also risk factors [7]. Similarly, in the analysis by Jäckel et al. in a group of patients after acute myocardial infarction and ICU stay >24 h, cardiac arrest was an independent predictor for delirium. In addition, age, dementia, alcohol abuse, hypotension, and leukocytosis were risk factors [17]. In contrast, a multivariable analysis by Falsini et al. in a group of ICU patients with acute cardiac disease did not identify cardiac arrest as a predictor. In this group of patients, risk factors included age, cognitive impairment, previous delirium, use of benzodiazepines and insulin, urinary catheterization, ventricular arrhythmias, hypernatremia, fever, and behavioral strategy [18].

A higher number of delirium days in post-cardiac arrest patients was associated with age and a longer time from the start of resuscitation to ROSC, which confirms the need to start high-quality CPR as soon as possible to restore perfusion [4]. An analysis by Rezar et al. found no gender difference in the incidence of delirium after CA [16].

While pre- and intra-cardiac arrest factors are nonmodifiable once the patient is in the ICU, there are many post-resuscitation elements of care that can be potentially modified. These include, among others, correction of hypoxia, metabolic disturbances and anemia, hyperthermia prevention or avoiding deep sedation [1].

Moderate sedation is recommended to prevent awareness and recollection, while avoiding the adverse effects associated with deep sedation, namely increased frequency of delirium and decreased survival [1]. Continuous infusion of sedative and analgesic drugs leads to accumulation and tolerance over time [8]. Practices to reduce exposure to psychoactive drugs are an important part of ICU care and strategies should be employed to reduce patient exposure as early as possible [1,7]. In a study by Needham et. al., low doses of ketamine were shown to reduce the incidence of delirium and its duration [9]. Foundraine et al. in their study compared the use in patients with out-of-hospital cardiac arrest of target temperature management (32–34 °C) together with intravenous sedation, with a modified method involving a combination of target temperature management (34–36 °C) with sevoflurane sedation. Analysis showed that the incidence of delirium in the group of patients sedated with sevoflurane was significantly lower (9/56, 16.1% vs. 25/67, 37.3%) [10]. The idea of volatile sedation in the ICU setting is not completely new. The devices for delivering it were described as early as 2005. Sevoflurane seems to be a very attractive alternative to intravenous sedation. It is metabolized in the liver and kidneys do not play a role in its metabolism and elimination, thus the post-resuscitation kidney injury and its influence on accumulation of active metabolites is not an issue here. After cessation of sedation its decreasing sedative effect is predictable and fast [29].

The subtype of delirium that occurred in most patients after CA was hypoactive: 90% of the patients in the Pollock et al. study had at least one day of hypoactive delirium [4]. Subsequently, more than half of the patients had a mixed delirium that lasted at least one day [5]. This is consistent with previous observations in ICU patients. Less than 5% of ICU patients experience purely hyperactive delirium, and the most common variety is hypoactive and mixed delirium [30,31]. On the other hand, according to the studies by Uguz et al. and Jäckel et al., in a group of patients after myocardial infarction the most common form was hyperactive delirium [17,20]. Patients were observed to have impaired orientation, attention and memory, psychomotor agitation, illusions, hallucinations, sleep−wake cycle disorders, and anxiety [16]. In a study by Keijze et al., most patients after CA experienced cognitive/perceptual impairment, psychomotor dysfunction, and impaired concentration and attention. A large number of patients also experienced extreme restlessness, disinhibition, emotional disturbances, and language disorders [2].

## 5. Conclusions

The incidence of delirium in ICU patients who have survived CA is high. Cardiac arrest is an additional predisposing factor for delirium. In cardiac arrest survivors, the occurrence of delirium prolongs the length of stay in the ICU and in hospital, adversely affecting functional outcomes. The most common type of delirium among this population is hypoactive delirium. A large percentage of patients manifested symptoms such as cognitive/perceptual impairment, psychomotor impairment, and impaired concentration and attention.

## 6. Implications for Practice

In the ICU setting, patients who survive cardiac arrest commonly receive sedative and analgesic medications as part of bundle care. The best way to treat delirium is to avoid it happening. Although presently there are no pharmacologic protocols for delirium prevention, given the altered pharmacokinetics and increased risk in this vulnerable population, there is a potential greater need to avoid deliriogenic medications. As the delirium treatment strategies are scarce, prevention with non-pharmacological means (e.i. early mobilization) seems a valuable option to pursue. There are some promising sedation protocols with not-so-novel medications, namely ketamine, dexmedetomidine and sevoflurane, that need to be explored for their potential of limiting delirium occurrence.

## Figures and Tables

**Table 1:** PCC framework, inclusion and exclusion criteria, search strategies.

	Inclusion Criteria	Exclusion Criteria
Participants (P)	Adult ICU patients	Children (>18 years) Non-ICU patients
Concept (C)	Delirium	No-delirium
Context (C)	Cardiac arrest	Other diseases
Types of evidence source	Observational, prospective, retrospective studies	Single-case report, cases report, letters to the editor
Years considered/time period	All evidence published in the past 10 years, period 2010–2020	Publications prior to 2010
Language	English	Other languages
Databases	MEDLINE (PubMed), Web of Science, EBSCO, Cochrane Library	Other databases
Keywords	Delirium, resuscitation, cardiac arrest	n/a
Additional search terms, with which the central search terms were combined	“ICU”, “intensive care”, “delirium”, “cardiac arrest”, “resuscitation”, “delirium after cardiac arrest”, “delirium after CPR”, “post-cardiac arrest” “incidence of delirium”	n/a

n/a—not applicable.

**Table 2:** JBI critical appraisal.

Author, year.	Q1	Q2	Q3	Q4	Q5	Q6	Q7	Q8	Q9	Q10	Q11
Rezar, R. et al. 2020 [16]	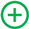	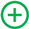	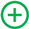	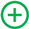	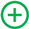	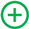	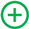	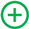	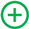	n/a	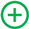
Keijze, H.M. et al. 2020 [2]	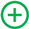	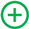	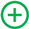	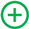	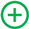	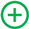	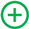	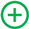	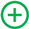	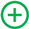	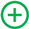
Jäckel, M. et al. 2020 [17]	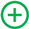	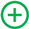	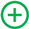	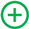	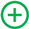	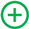	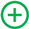	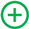	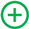	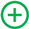	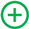
Falsini, G. et al. 2018 [18]	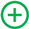	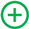	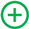	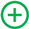	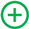	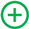	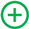	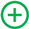	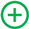	n/a	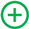
Pollock, J.S. et al. 2016 [4]	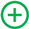	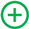	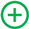	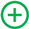	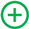	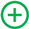	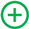	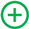	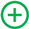	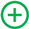	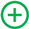
Pauley, E. et al. 2015 [19]	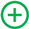	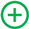	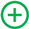	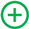	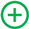	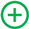	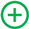	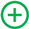	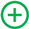	n/a	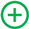
Uguz, F. et al. 2010 [20]	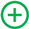	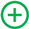	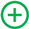	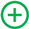	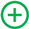	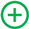	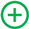	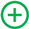	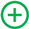	n/a	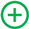

**Table 3:** Tabular presentation of qualitative findings for a scoping review.

Author, Year	Study Design	Participants	Delirium Assessment Tool	No. of Delirium Incidents after CA	Findings
Rezar, R. et al. 2020 [16]	A prospective analysis	Adult patients hospitalized at a medical ICU after CPR	No data	24/106 (23%)	-Delirium occurred in 22.6% of patients after CA -There was no statistically significant difference in the incidence of delirium after CA in males and females
Keijze, H.M. et al. 2020 [2]	An ad hoc analysis of a multicenter prospective cohort study	Patients with recovery of consciousness, who survived until hospital discharge	Psychiatric consultation (DSM-V criteria)	47/141 (33%)	-Delirium is common after CA -Delirium leads to longer hospitalization and poorer outcome
Jäckel, M. et al. 2020 [17]	A retrospective study	Patients (ICU) hospitalized for MI treated with coronary angiography	RASS and NuDesc	15/68 (22%)	-CA was an independent predictor of delirium
Falsini, G. et al. 2018 [18]	A prospective, observational cohort study	CICU patients	RASS and CAM	9/111 (8%)	-CA was not a predictor of delirium
Pollock, J.S. et al. 2016 [4]	A retrospective observational study	Patients (CICU) treated with therapeutic hypothermia after cardiac arrest	RASS and CAM-ICU	107/107 (100%)	-High prevalence of delirium during the ICU stay in patients treated with TH after cardiac arrest -Most of the episodes of delirium were hypoactive -Older ages, longer times from initiation of CPR to ROSC were associated with increased duration of delirium.
Pauley, E. et al. 2015 [19]	A retrospective study	Patients admitted to CICU with a primary cardiovascular diagnosis	RASS and CAM-ICU	21/120 (18%)	-Patients admitted after cardiac arrest were more likely to be CAM-ICU positive
Uguz, F. et al.2010 [20]	A retrospective study	Patients with acute MI admitted to the CICU	Psychiatric assess (DSM-IV-TR criteria	3/12 (25%)	-CA during MI was an independent predictor of development of delirium

CPR—cardiopulmonary resuscitation; ICU—intensive care unit; CICU—cardiac intensive care unit; CA—cardiac arrest; MI—myocardial infarction; TH—therapeutic hypothermia; ROSC—return of spontaneous circulation; NuDesc—Nursing Delirium Screening Scale; DSM—Diagnostic and Statistical Manual of Mental Disorders; CAM-ICU—confusion assessment method for the ICU; RASS—Richmond Agitation and Sedation Scale.

## Data Availability

The authors declare that the data of this research are available from the correspondence author on request.
